# How appropriate are ChatGPT-5’s answers to acne questions? A category-based evaluation by dermatologists

**DOI:** 10.1097/MD.0000000000050664

**Published:** 2026-09-18

**Authors:** Nazli Caf, Defne Özkoca, Tuğba Kevser Üstünbaş Uzunçakmak, Zekayi Kutlubay

**Affiliations:** a Department of Dermatology and Venerology, Faculty of Medicine, Istanbul Atlas University, Istanbul, Türkiye; b Department of Dermatology and Venerology, Health Sciences University, Başakşehir Çam and Sakura City Hospital, Istanbul, Türkiye; c Department of Dermatology and Venerology, Faculty of Medicine, Istanbul University-Cerrahpaşa, Istanbul, Türkiye.

**Keywords:** acne vulgaris, artificial intelligence, dermatology, patient education

## Abstract

Online conversational tools have become increasingly common sources of health information, with many patients seeking acne advice through such platforms. Although they may enhance awareness and encourage early consultation, their medical reliability remains uncertain. Because acne is one of the most frequently searched dermatologic conditions, verifying the accuracy of automated responses is crucial to prevent misinformation. This study aimed to evaluate ChatGPT-5 responses to frequently asked acne questions in dermatology practice and to determine category-specific strengths and limitations based on blinded expert assessment. The analysis focused on identifying error patterns with potential implications for patient education and clinical safety. Twenty-five commonly asked patient questions were classified into 4 categories: skin care routine, nutrition and lifestyle, diagnosis and general information, and treatment and medications. Each question was entered into the publicly available ChatGPT-5 interface in 3 independent sessions, yielding 75 unique responses. Three dermatologists independently rated each response as “appropriate” or “inappropriate,” and reasons for rejection were categorized using a predefined 9-item list. Of 75 responses, 49 (65.3%) were appropriate. Appropriateness was highest for skin care (83.3%) and nutrition (77.8%) and lowest for treatment (50.0%) and diagnosis (33.3%). Inter-rater agreement was low. The most frequent reasons for inappropriateness were misleading information and overly technical language, followed by lack of risk disclosure. ChatGPT-5 may provide helpful introductory information for patients; however, diagnostic and treatment guidance often lacked clarity and safety detail, underscoring the need for dermatologist oversight.

## 1. Introduction

Patients’ search for quick and understandable information from online sources has made question-and-answer-based chat interfaces visible in healthcare communication, and they have become quite popular recently. In a comparison published in the Journal of the American Medical Association Internal Medicine, responses provided by chatbots to patient questions on public social media platforms scored higher than physician responses in terms of both quality and empathy; however, the study did not measure accuracy as a primary outcome and recommended caution in interpreting the results from a clinical safety perspective.^[1]^ The same point was emphasized in the popular press: Chat tools can facilitate patient communication, but physician supervision is necessary to ensure accuracy, timeliness, and security.^[1]^

Some early reports in dermatology have shown that most of chat generative pre-trained transformer (GPT, ChatGPT)’s responses to patient questions can be considered “appropriate (A)”; however, inconsistencies arise, particularly in topics such as diet/lifestyle, alternative suggestions, and complex clinical decisions. Ferreira et al reported 88% appropriateness based on 3 responses from 3 separate sessions for each question and the majority decision of 3 dermatologists; however, incorrect or unsupported statements were identified in certain subject areas.^[2]^ Subsequent studies elaborated on the table: Usefulness varied among free chat tools; readability generally remained at the high school level; and diagnostic accuracy was low in certain subfields.^[3–5]^ In particular, it has been reported that ChatGPT-5’s sensitivity may be insufficient in critical tumor lesions such as malignant melanoma and basal cell carcinoma; these findings constitute a serious and clear warning against the use of chat interfaces for diagnostic purposes.^[3,4]^ Additionally, in a comparative study conducted for hidradenitis suppurativa (HS), although ChatGPT-4 was rated highly by patients, dermatologists were preferred as the ultimate source of information.^[6]^

However, as seen in non-dermatology topics, chat interfaces can provide a practical starting point for producing patient education drafts; shortcomings tend to be concentrated in areas that can be edited, such as lack of evidence attribution, failure to clearly indicate risks, and language that is overly technical or detailed. In readability studies in dermatology, content is often reported at a 10th-grade level; it has been shown that lowering it to the targeted health literacy threshold (e.g., 6th–8th grade) is possible with conscious guidance and expert editing.^[1,5,7]^ For educational content to be used in clinical practice, dermatologist supervision, guideline-based updating, and simplification of language are essential requirements.^[1,5,8]^

In the current literature, there are limited studies conducted with a Turkish questionnaire specific to acne, in which responses are obtained from 3 separate sessions for each question and a majority decision is reached through blind evaluation by 3 dermatologists. In addition, there is little data that quantitatively explains why “inappropriate (I)” responses are rejected using a standard 1 to 9 explanation list. This study collects the 25 most frequently asked acne questions in the outpatient clinic under 4 categories (skin care routine, nutrition & lifestyle, diagnosis & general information, treatment & medications) and bases its findings on the majority decision of 3 dermatologists who independently and *blindly* evaluate 3 responses for each question. The reasons for responses classified as “I” are then quantitatively reported under 1 to 9 explanation headings through a wrap-up consolidation by a fourth dermatologist. Thus, this study not only reveals compliance rates but also error patterns, providing a practical framework for identifying areas where patient education texts can be improved.^[2–5,8]^

## 2. Materials and method

### 2.1. Work design

The study was conducted cross-sectionally and observationally. The aim was to evaluate ChatGPT responses to the most frequently asked patient questions related to acne by 3 dermatologists in the form of “A/I” and to quantitatively document the reasons for rejecting responses deemed “I” using a list of 1 to 9 explanations.

We used ChatGPT (GPT-5, OpenAI) solely as a data source to obtain responses in August 2025, as detailed in the Methods; no generative editorial work was performed,

### 2.2. Question sets and categories

A set of 25 questions most frequently asked by acne patients in the outpatient clinic was compiled by a fourth dermatologist – who did not participate in querying ChatGPT – and classified into 4 categories: skin care routine, nutrition and lifestyle, diagnosis and general information, and treatment and medications. The formulation of each question was standardized, and the same text was used in all sessions. Table 1 provides the question sets in detail.

**Table 1:** Questions directed to the chat interface.

Category	Question number	Question
Nutrition & lifestyle	3	Do sugary and fatty foods worsen acne?
Nutrition & lifestyle	4	Do milk and dairy products cause acne?
Nutrition & lifestyle	9	Is there a relationship between acne and stress?
Nutrition & lifestyle	15	Does sunbathing, using a solarium, and/or swimming in the sea help with acne?
Nutrition & lifestyle	17	Are probiotics or supplements beneficial for acne?
Nutrition & lifestyle	23	Would completely eliminating gluten help improve my acne?
Skin care routine	1	Will washing my face more frequently clear my acne faster?
Skin care routine	6	Would adding products such as toner, mask, or scrub to my routine help with acne?
Skin care routine	11	Is it harmful to cover pimples with concealing makeup products?
Skin care routine	14	Does using sunscreen worsen my acne?
Skin care routine	19	My acne is gone but I still have enlarged pores, can they be reduced?
Skin care routine	20	Can retinoic acid (tretinoin) and AHA/BHA be used at the same time?
Skin care routine	24	Would using a moisturizer make my acne worse?
Skin care routine	25	Should everyone with acne perform double cleansing?
Diagnosis & general information	5	Are blackheads and acne the same thing?
Diagnosis & general information	7	Is it normal to have acne outside of adolescence?
Diagnosis & general information	10	If I have acne, should I have hormone tests done?
Treatment & medications	2	Why is popping pimples harmful?
Treatment & medications	8	Can acne be treated with natural methods (masks, herbs)?
Treatment & medications	12	Does oral isotretinoin treatment used for acne cause infertility?
Treatment & medications	13	My pimples are gone but redness and spots remain, what should I do?
Treatment & medications	16	Which treatments are most effective for acne scars?
Treatment & medications	18	Do acne medications thin out the skin
Treatment & medications	21	My face becomes very red and peels with acne creams. Is this normal? What can I do?
Treatment & medications	22	Which acne treatments are safe during pregnancy?

AHA = alpha hydroxy acid, BHA = beta hydroxy acid.

### 2.3. Obtaining responses

Questions were asked in 3 separate sessions between August 8 and August 16, 2025, on the publicly available ChatGPT (GPT-5 web interface), with no transfer of previous session content. This resulted in 3 independent responses for each question, for a total of 75 responses. No additional system instructions were provided; responses were recorded without any editing.

### 2.4. Evaluators and blinding

Three dermatologists, consisting of 2 associate professors and 1 professor, classified each response as “A” or “I” without seeing each other’s scores (blind). The majority decision (≥ 2 same votes) for each response was recorded as the primary decision. The evaluators’ notes were used only to support the rationale for the “I” decision; the decision itself was given in a binary (A/I) format.

Consolidation of explanations for “I” responses (1 to 9)

Responses that remained “I” according to the majority were wrapped up by the fourth dermatologist and consolidated with a list of 1 to 9 explanations consistent with the reasons reported by the first 3 evaluators; the fourth evaluator dermatologist did not produce an independent new decision and did not change the majority decision. (See Table 2 for the list of explanations used and their frequencies.)

**Table 2:** Appropriateness by category.

Category	Appropriate	Inappropriate	Total	Appropriate%	Lower 95% CI	Upper 95% CI
Nutrition & lifestyle	14	4	18	77.8	54.8	91.0
Skin care routine	20	4	24	83.3	64.1	93.3
Diagnosis & general information	3	6	9	33.3	12.1	64.6
Treatment & medications	12	12	24	50.0	31.4	68.6

CI = confidence interval.

### 2.5. Outputs

- Primary outputs: Adequacy rate (A/75) and 95% confidence interval (CI) for all responses.

- Secondary outputs:
  - Suitability rates by category and differences between categories,
  - Inter-rater agreement (Fleiss κ),
  - Distribution of explanations for “I” responses, 1 to 9,
  - Planned sensitivity analyses,
  - Answer sequence effect (Answer 1/2/3; 3 × 2 table),
  - Question-based success: If ≥2/3 answers are correct for each question, the question is considered “successful” with a ratio of 25 items and 95% CI.

### 2.6. Statistical analysis

The analyses were performed using Statistical Package for the Social Sciences 27.0 (International Business Machines Corporation) 27.0 Statistics. Since there were no continuous variables, parametric tests were not used.

- Ratios are given as percentages; the Wilson method was used for the 95% CI.
- Inter-category comparison: Pearson chi-square test (2-tailed, *P* < .05). Cell contributions were interpreted with standardized residuals when necessary; the Bonferroni/Holm approach was planned for multiple test correction in pairwise comparisons.
- Inter-rater agreement: Fleiss kappa coefficient was calculated for 3 raters and 2 categories (A/I):
- κ = Pˉ − Pe1 − Pe\kappa = \frac{\bar{P} − P_e}{1 − P_e}κ = 1 − Pe Pˉ − Pe
- Here, $\bar{P}$ is the observed mean agreement, and *P*_*e*_ is the expected fit based on marginal probabilities. In this series, κ = −0.227, standard error ≈ 0.059, *z* ≈ −3.83, and *P* <.001 were found. The negative value suggests that the observed *agreement* is lower than expected by chance; the aggregation of the gray areas of the binary classification toward “inappropriate” and the amplification of interpretation differences in questions at the diagnosis/treatment boundary indicate a weakening of the *agreement*.
- Sensitivity analyses: for response order effects, a 3 × 2 chi-square test was performed, and for question-based success, a ratio and 95% CI (Wilson) were calculated based on 25 items.

### 2.7. Ethical statement

The study does not include human participant data. Questions were posed by the researcher in the role of a patient on a public chat interface; the text responses obtained were evaluated by a panel of experts. Ethical committee approval was not required according to local regulations; exemption conditions were met.

## 3. Results

General appropriateness results are summarized in Table 2. Of the total 75 responses, 49 were classified as “(A)” and 26 as “(I)”; the overall appropriateness was 65.3% (95% CI 54.1–75.1).

The results regarding the appropriateness by categories are summarized in Table 3. The appropriateness rates were 83.3% (20/24) for skin care routine, 77.8% (14/18) for nutrition & lifestyle, 50.0% (12/24) for treatment & medications, and 33.3% (3/9) for diagnosis & general information. The difference among categories was significant (*χ*^2^(3, N = 75) = 11.22; *P* = .0106). The effect size was Cramér’s V = 0.387 (moderate level). Residual patterns showed a higher-than-expected contribution of “(A)” responses in the “skin care” and “nutrition” categories, and a higher-than-expected contribution of “(I)” responses in the “diagnosis” and “treatment/medications” categories.

**Table 3:** Explanation distribution (1 to 9) of responses classified as “Inappropriate (I)”.

Explanation Code	Frequency	Explanation Text (English)
1	10	Contains misleading information
2	2	Response is filled with unnecessary details and off-topic
3	5	Response is too technical and not understandable for the patient
4	3	Response is misleading; risks are not specified
5	1	Response contains contradictory information
6	1	Response contains nonscientific recommendations
7	1	Partially correct; does not fully answer the patient’s question
8	2	Response is incomplete and important points are omitted
9	1	Response contains nonscientific data

Inter-rater agreement. The agreement among the 3 dermatologists was found to be Fleiss κ = −0.227 (standard error is approximately 0.059; z is approximately −3.83; *P* < .001). The marginal probabilities were p(A) = 0.629 and p(I) = 0.371; the observed mean agreement was P̄ = 0.427, and the expected agreement was P_e_ = 0.533. The negative κ suggests that the binary classification tends to collapse gray areas toward “(I)” and that items at the diagnosis/treatment boundary increase interpretation differences, resulting in observed agreement being lower than expected by chance.

Explanations for “(I)” responses. For the 26 responses classified as “(I)” by the majority, the distribution of explanations 1 to 9 was as follows:

1: 10; 2: 2; 3: 5; 4: 3; 5: 1; 6: 1; 7: 1; 8: 2; 9: 1.

The most common reasons were 1: “misleading information” and 3: “too technical, not understandable for the patient,” followed by 4: “risks not specified.” (Table 3).

Sensitivity analyses:

- Response order effect: No significant difference in appropriateness was observed among responses 1/2/3 (chi-square, *P* = .662).
- Question-level success: Using the definition “a question is successful if ≥ 2/3 responses are “(A),” success was observed in 17/25 questions (68.0%; 95% CI 48.4–82.8); the pattern was consistent with the main analysis (higher in skin care and nutrition, lower in diagnosis and treatment).
- Overall appropriateness: 49/75 = 65.3% (95% CI 54.1–75.1). Difference among categories: *χ*^2^ = 11.22, df = 3, *P* = .0106; Cramér’s V = 0.387.

Detailed responses generated by the model and rater evaluations are provided in the online repository (Table S1, Supplemental Digital Content 1).

## 4. Discussion

In this study, we demonstrated that 2-thirds of ChatGPT’s responses to a patient-facing acne-related questions were deemed as “(A)” by the majority of dermatologists; appropriateness was high in the skin care and nutrition categories but markedly lower in the diagnosis and particularly in the treatment/medication categories. The main reasons for “(I)” decisions were misleading information and overly technical/unintelligible language, followed by lack of risk disclosure. This pattern suggests that clear risk–benefit explanations, contraindications, dosage/follow-up details, and patient-friendly language are essential for clinical safety.

Previous dermatology studies have reported high appropriateness rates (88%) for GPT-5–based ChatGPT across a wide range of topics; however, it was emphasized that the errors largely clustered around lack of evidence, incompleteness, and misleading information.^[2]^ In our acne-focused, Turkish-language, and category-based design, the lower appropriateness rate is consistent with the clinical borderline nature of the question set (diagnosis and treatment details), differences in language/context, and the strictness of the panel. Moreover, our design quantitatively captured the reasons for “(I)” decisions using a standardized 1 to 9 explanation list, thereby concretizing areas for improvement. In this respect, it not only measures performance but also provides a targeted roadmap for optimization.

A recent comparative study reported that in the free versions (ChatGPT 3.5, Bard, Bing AI), patient-facing utility was variable, and that chatbots demonstrated the greatest potential when used under dermatologist supervision.^[5]^ Our findings, particularly the lower appropriateness in the treatment/medication category and the deficiencies in risk disclosure, support this conclusion; they indicate that while chat-based interfaces can be useful for draft generation, they may fall short for final content without expert oversight. Of course, conducting studies at different time points (as versions may change) and with a broader range of questions will be important to fully validate this conclusion.

Studies conducted in the field of skin cancer using artificial intelligence (AI) have reported that ChatGPT-4/4o is not reliable in terms of sensitivity and specificity for diagnostic accuracy, particularly in critical areas such as differentiating malignant melanoma from basal cell carcinoma/squamous cell carcinoma.^[3,4,7]^ When our questions approached diagnostic decision-making details, the appropriateness rate decreased and the frequency of “misleading/incomplete” explanations increased, which is consistent with the literature and reaffirms that diagnostic use is not appropriate. Indeed, Lewandowski et al also noted that despite patients evaluating AI responses more positively, dermatologists remain the primary source of information for definitive clinical decisions.^[6]^ We advocate that expert consultation is essential when it comes to diagnosis.

In our study, the frequent occurrence of the “too technical/unintelligible” explanation is also consistent with readability findings: In a study conducted on HS, ChatGPT responses were found to be approximately at a college junior–senior reading level, well above the recommended 6th to 8th grade target level for health literacy.^[8]^ In our results, the need to simplify patient-facing texts is also evident. In clinical practice, shortening chatbot outputs, simplifying technical terminology, and adding sources/evidence could serve as an effective correction pathway. Similarly, in HS-focused studies, while AI responses appear positive in terms of language and relational qualities, expert review is recommended prior to clinical use due to deficiencies in content standardization and referencing.^[6]^ We encourage further studies in this area.

The negative κ (−0.227) for inter-rater agreement is noteworthy. This finding can be reasonably interpreted based on 3 factors: the binary framework “(A/I)” inevitably pushes gray areas to 1 side, most often toward “(I),” causing intermediate nuances to be lost; since part of the question set lies at the diagnosis/treatment boundary, interpretive differences based on clinical nuance are expected; no calibration session was held, panel members may have weighed “safety” and “adequacy” differently. For these reasons, the negative κ does not mean that the chatbot is “always wrong”; rather, it reflects differing professional thresholds under a strict binary assessment. In the future, the use of intermediate categories such as “partially appropriate,” pre-assessment training, and a broader panel could be expected to yield a more balanced κ. This interpretation is supported by the classic Landis–Koch classification.

This study has major strengths. First, it provides one of the earliest dermatology-focused evaluations of ChatGPT-5, offering timely data for a new model whose clinical performance has not yet been comprehensively examined. Using an acne-specific question set derived directly from routine outpatient encounters ensures that the content reflects real patient concerns rather than hypothetical scenarios. Each question was presented in 3 independent sessions, yielding 75 unique responses and allowing an assessment of response variability that has been largely overlooked in earlier work. The blinded evaluation by a panel of 3 dermatologists, combined with majority-based classification, enhances the reliability of the judgments and reduces individual reviewer bias. A further strength is the structured analysis of “I” responses using a predefined 9-item explanation list, which permits a systematic characterization of error patterns and clarifies which aspects of patient-facing information require attention. Finally, the category-based design (covering skin care, lifestyle, diagnosis, and treatment) provides a nuanced understanding of where the model performs well and where potential safety concerns emerge, offering practical guidance for clinicians and informing future refinement of chat-based patient education tools.

The limitations of this study include the sample being restricted to acne, which may limit generalizability to other dermatological diseases. The evaluation was binary, and intermediate levels of accuracy were not quantified. The responses are products of the web interface dated August 8 to 16, 2025; model updates may alter performance. In addition, the negative κ reflects the rigidity of the method and threshold differences among the panel; therefore, the results should be interpreted based on the “majority decision.” As Ferreira et al have noted, such studies are time-sensitive and require iterative validation.^[2]^

From a practical standpoint, in the field of acne, chat-based interfaces may be useful for providing “initial information” and drafting patient education materials; however, they should not be used in diagnostic contexts or in treatment/medications categories without visible risk disclosures and up-to-date guideline references. Institutions could develop standardized acne information templates (indications, dosage, contraindications, adverse effects, follow-up) and integrate them with chatbot outputs to accelerate the process; nevertheless, the final content must always be approved by a dermatologist. As demonstrated in the Journal of the American Medical Association study, chat-based interfaces can enhance text quality and empathy; however, this does not replace the need for accuracy and safety oversight.^[1]^

Future studies should include a broader question set and a multicenter sample; use intermediate categories (partially appropriate) with multi-level scoring; perform agreement analysis after calibration sessions; apply Generalized Estimating Equations (-based) models to robustly estimate category effects while accounting for within-question correlation; and adopt experimental designs testing the impact of interventions aimed at reducing readability and comprehensibility to the target grade level. This study is important as it is the first conducted in this field from Türkiye and contributes to the global literature.

## Author contributions

**Conceptualization:** Nazli Caf.

**Data curation:** Nazli Caf.

**Formal analysis:** Tuğba Kevser Üstünbaş Uzunçakmak.

**Investigation:** Nazli Caf, Defne Özkoca, Tuğba Kevser Üstünbaş Uzunçakmak, Zekayi Kutlubay.

**Methodology:** Nazli Caf, Zekayi Kutlubay.

**Project administration:** Nazli Caf.

**Supervision:** Nazli Caf.

**Validation:** Tuğba Kevser Üstünbaş Uzunçakmak.

**Writing – original draft:** Nazli Caf.

**Writing – review & editing:** Nazli Caf, Defne Özkoca, Zekayi Kutlubay.


