# Supplementary material for: How appropriate are ChatGPT-5’s answers to acne questions? A category-based evaluation by dermatologists
Source: Medicine (Baltimore). 2026 Sep 18;105(38):e50664. doi: 10.1097/MD.0000000000050664 (PMC13593273; doi:10.1097/MD.0000000000050664)
Supplement: Supplementary file 1 [file medi-105-e50664-s001.docx]

**Supplementary Table 1- Questions Directed to the Chat Interface and the Three different Answers Obtained in Detail**

| Question No | Question | Answer 1 | Answer 2 | Answer 3 |
| --- | --- | --- | --- | --- |
| 1 | Will washing my skin more often make my acne clear up faster? | It would not be accurate to say that washing your skin more often will necessarily make acne clear up faster—in fact, it can sometimes make acne worse. Washing your face twice a day, morning and evening, with a gentle cleanser can help reduce acne by removing excess oil, dirt, and dead skin cells. Washing more frequently (3–4+ times a day) can strip too much of the skin's natural oils, which may cause the skin to produce more oil. Excess oil can clog pores and increase acne. Frequent washing can also irritate the skin, especially when combined with harsh cleansers or exfoliating products, and this may worsen inflamed pimples. | No, washing your skin more often does not make acne go away faster; in fact, it can make it worse. Washing your face twice a day (morning and evening) with a gentle cleanser is generally enough. More frequent washing can disrupt the skin's natural oil balance, dry it out, and cause irritation. This may prompt the skin to produce more oil and make it easier for pores to become clogged, creating conditions for new breakouts. Harsh soaps, very hot water, and aggressive cleansers can also weaken the skin barrier. The best approach is consistent but gentle cleansing: wash morning and evening with a suitable cleanser, and cleanse after sweating or wearing makeup. In short, acne is better managed by washing more thoughtfully, not more often. | Washing your skin more frequently does not make acne heal faster. On the contrary, over-washing can damage the skin barrier, cause dryness and irritation, and lead the skin to produce more oil, which may increase breakouts. In general, washing the face twice a day—morning and evening—with a gentle cleanser is sufficient. More than that is not recommended because it may disrupt the skin's natural balance. It is also important to use products suited to your skin type and formulated for acne-prone skin. If your acne is widespread or severe, it is healthier to see a dermatologist and develop a professional treatment plan. If you wish, we can also review the products you use or discuss skincare options suitable for your skin type. |
| 2 | Why is popping pimples harmful? | It increases the risk of infection: your fingers or nails may carry bacteria, and when you squeeze a pimple you can push those bacteria into an open pore. When you squeeze an inflamed pimple, the bacteria and pus inside may also spread into deeper layers of the skin, causing larger and more painful inflammation. It increases the risk of scarring: squeezing causes trauma to the skin, which can result in permanent acne scars, especially in people with sensitive skin or those who frequently pick at pimples. It can also cause discoloration: damage after squeezing may lead to hyperpigmentation, particularly in darker or olive-toned skin. Finally, inflammation can spread into surrounding tissue and contribute to the formation of additional pimples. | Popping pimples is harmful because it can push inflammation deeper into the skin and into surrounding tissues rather than simply bringing it to the surface. Fingers and nails are often not completely clean, so new bacteria may be introduced and the infection can worsen. Squeezing also damages the structure of the skin, delays healing, and may leave dark marks or permanent depressed scars. Sometimes the inflamed material is not fully expelled and instead spreads under the skin, which can lead to larger, painful cystic pimples. Healthy tissue around the traumatized area can also be damaged. For these reasons, leaving pimples alone and using appropriate acne treatments is a much better approach for healthier healing with less risk of scarring. | Popping pimples is generally harmful to skin health because it can cause bacteria inside the pimple to spread into deeper layers of the skin or nearby tissue. This may increase inflammation, make the pimple larger and more painful, and prolong healing. Squeezing also causes skin trauma, which can result in permanent scars, dark marks, or depressed scars. If the hands are not clean enough, new bacteria can be transferred to the skin and increase the risk of infection. Although popping a pimple may seem to reduce it in the short term, it can damage the skin and lead to more serious cosmetic problems in the long term. It is therefore best not to pick or squeeze pimples and, when necessary, to treat them with appropriate products or dermatologic support. |
| 3 | Do sugary and fatty foods increase acne? | Yes, sugary and fatty foods may increase acne in some people—especially if your skin is already acne-prone. However, the effect varies from person to person, so eating chocolate does not automatically cause pimples in everyone. Research and dermatologic observations suggest that some foods may be associated with acne. High-glycemic foods such as white bread, white rice, sweets, and soft drinks can rapidly raise blood sugar and insulin. Higher insulin levels may increase androgen activity, oil production, and pore clogging, potentially leading to more breakouts. Fatty and processed foods such as fast food, fried foods, chips, crackers, and packaged pastries often contain trans and saturated fats and may increase inflammation. Milk chocolate has also been linked to acne in some studies because it contains both dairy and sugar; dark chocolate may have less of this effect, although individual responses differ. | Whether sugary and fatty foods worsen acne has been debated for years, but more recent research suggests that high-glycemic foods and some unhealthy fatty foods may contribute to acne. Sugary foods and refined carbohydrates can raise insulin and IGF-1 levels, which may stimulate the sebaceous glands and increase keratin production, making pore blockage and inflammation more likely. Fatty foods do not transfer oil directly to the skin, but highly processed foods rich in unhealthy fats can contribute to excess calorie intake and obesity risk, indirectly affecting hormonal balance and potentially worsening acne. Healthy fats such as olive oil, fish oil, and avocado are beneficial for general skin health and are not known to increase acne. A balanced, low-glycemic diet rich in whole foods may therefore support acne management, while limiting excessive sugar and unhealthy fats may have a positive effect on the skin. | Yes, sugary and fatty foods can worsen acne in some people, although this is not true for everyone because skin responses vary. Foods high in refined sugar can rapidly raise blood glucose and insulin, which may increase androgen activity and sebum production. Sebum, the oil produced by the skin, can contribute to clogged pores and acne. Similarly, fast-food-style meals high in saturated fat may promote inflammation and disrupt skin balance. Because these foods are often low in fiber, they may also negatively affect gut health and indirectly influence skin problems. Still, metabolism differs between individuals: some people may eat these foods often without obvious worsening, while acne-prone or sensitive skin may react to even small dietary changes. Observing your own skin and seeking professional advice when needed is the best approach. |
| 4 | Do milk and dairy products cause acne? | Yes, milk and dairy products may trigger acne in some people—particularly skim or low-fat milk and some processed dairy products. However, this does not apply to everyone. Some people consume dairy without any problems, while others notice a clear increase in breakouts. Personal observation is therefore important. Skim milk and certain dairy products, especially UHT and flavored milks, have been associated with acne. One possible reason is the naturally occurring hormone-like compounds and growth factors in milk, such as IGF-1, which may stimulate sebaceous glands, increase oil production, and contribute to clogged pores. You do not necessarily need to eliminate dairy completely; you can reduce it and observe your skin. During acne-prone periods, alternatives such as almond, oat, or coconut milk can be tried instead of skim milk. | The relationship between dairy and acne has been studied for a long time. Recent research suggests that particularly skim milk and some dairy products may be associated with acne. Milk may influence insulin-like pathways through its hormones and growth factors, potentially increasing sebum production and pore blockage. It may also raise IGF-1, which can contribute to inflammation and pimple formation. Interestingly, some studies have found a stronger association with skim or low-fat milk than with whole milk, possibly because hormone-related components are more concentrated in skim milk. There is less strong evidence that fermented dairy products such as yogurt and kefir have the same effect, and they may even support gut health. Overall, dairy can worsen acne in some people but not everyone. If you notice a connection, reducing dairy for a period and observing your skin may be useful. | Milk and dairy products may trigger acne or make existing breakouts more severe in some people, but this does not happen to everyone. Cow's milk in particular may affect levels of IGF-1, which can increase oil production, promote clogged pores, and create conditions favorable to acne. Naturally occurring or added hormones in dairy may also contribute to hormonal effects, especially during adolescence. On the other hand, fermented products such as yogurt and kefir may support the gut microbiome and could have different effects on the skin. The impact depends on the type and amount of dairy as well as individual genetics. If you notice more acne after consuming dairy, you can temporarily reduce or avoid it and monitor the change. A dermatologist or nutrition professional can help if you want a more definite assessment. |
| 5 | Are blackheads and acne the same thing? | No. Blackheads and acne are not exactly the same. A blackhead is a type of acne lesion called a comedone, but it is non-inflammatory. Acne is a broader condition that includes both inflammatory and non-inflammatory types of lesions. | Blackheads and acne are often confused, but they are not the same, although blackheads can be considered one type or stage of acne. A blackhead is medically called an open comedone. It forms when sebum and dead skin cells collect in a pore and the material darkens after contact with oxygen. It is a non-inflammatory acne lesion. Acne is a broader term that includes blackheads, whiteheads (closed comedones), inflammatory papules and pustules, and deeper lesions such as nodules and cysts. In other words, every blackhead is an acne lesion, but not every acne lesion is a blackhead; acne can appear in many different forms, including non-red and non-painful lesions. | Blackheads and acne are related but not identical. Blackheads are 'open comedones' that form when pores fill with oil and dead skin cells and remain open at the surface; the contents oxidize on contact with air and turn dark. They are common on oily areas such as the nose, chin, and forehead. Acne is a broader skin problem and may also involve bacterial growth and inflammation, which can create red, swollen, or painful lesions. A blackhead may be considered an early or non-inflammatory form of acne, but not every blackhead will turn into an inflamed pimple. Good cleansing and regular skincare can help limit blackheads and reduce the chance of further clogging. |
| 6 | Would adding toner, masks, or scrubs to my routine help acne? | Yes, toner, masks, and scrubs can help acne when they are used correctly and are suitable for your skin type. They may help clean pores, balance excess oil, and remove dead skin cells. However, they must be used carefully because choosing the wrong products or using them too often can irritate the skin and worsen acne. Physical scrubs in particular can cause micro-injury when used too frequently or aggressively. Alcohol-based toners may dry the skin and trigger more oil production. A gentle mask once or twice a week and a well-formulated toner can support skin health when used as part of a balanced routine. | Adding toner, masks, or scrubs may help acne, but choosing and using the right products is important; otherwise, irritation may make the condition worse. Alcohol-free toners containing ingredients such as salicylic acid or niacinamide can help clear pores and balance excess oil, while highly drying alcohol-based toners can damage the skin barrier. Clay masks once or twice a week may absorb excess sebum, but overuse can cause dryness and irritation. Physical scrubs should be used cautiously because large or rough particles can scratch inflamed acne, spread irritation, and increase the risk of marks. Chemical exfoliants containing low concentrations of AHA or BHA may be a gentler and more effective alternative. Products should be selected according to skin type and acne severity and used in moderation. | Toner, masks, and scrubs can sometimes support acne care, but how often they are used, what they contain, and your skin type all matter. Toners with salicylic acid or niacinamide may help clear pores and regulate oil, whereas harsh alcohol-based toners can dry and irritate the skin. Clay masks may absorb excess oil but can weaken the skin barrier when overused. Mechanical scrubs are generally not recommended for sensitive or actively inflamed acne because their particles can cause micro-damage; gentler chemical exfoliants containing salicylic or glycolic acid may be more suitable. The best approach is to first establish a basic routine appropriate for your skin type and then add extra products slowly and carefully as needed. |
| 7 | Is it normal to have acne beyond adolescence? | Yes, having acne outside adolescence is completely normal. Acne often begins during puberty because of hormonal changes, but many people continue to experience it as adults, including in their 20s, 30s, and even 40s. In women especially, the menstrual cycle, birth-control pills, pregnancy, and conditions such as PMOS can trigger acne. Stress, irregular sleep, diet, certain medications, and unsuitable skincare products can also contribute. Adult acne is therefore not unusual and affects many people. It can be treated; the important thing is to identify and address the relevant causes. | Acne outside adolescence is quite common and is generally considered normal. Although acne is especially common during puberty because of increased hormone levels, it may continue or begin after the twenties. In women, menstrual cycles, pregnancy, starting or stopping hormonal contraceptives, and polycystic ovary syndrome can trigger breakouts. Stress, poor sleep, diet, some medications, and unsuitable skincare habits may also contribute. Adult acne can sometimes be deeper and more inflammatory and may concentrate on the lower face and jawline. If it occurs, consulting a dermatologist can help identify contributing factors and determine appropriate treatment. | Yes, acne can occur well beyond adolescence and is very common. Many people continue to have acne after puberty and even into their thirties and forties. This is often called adult acne and is especially common in women because hormonal fluctuations related to the menstrual cycle, contraception, pregnancy, or menopause can influence sebum production. Stress, irregular sleep, diet, medications, unsuitable cosmetics, genetics, and naturally clog-prone or oily skin can also play a role. Adult acne is therefore not unusual, and treatment is usually possible once contributing factors are properly assessed. Persistent or widespread acne may warrant evaluation by a dermatologist, who can tailor treatment and, when appropriate, consider hormonal testing. |
| 8 | Can acne be treated with natural methods such as masks or herbs? | Natural methods can support treatment when acne is mild, but they may not be enough on their own. When used appropriately, some plant-based masks and natural ingredients may help balance oil, reduce inflammation, and clean pores. Tea tree oil, for example, may help mild pimples because of its antibacterial properties. Honey, aloe vera, and green tea may soothe the skin and support healing. However, natural approaches alone are usually insufficient for moderate or severe acne, cystic lesions, or acne that has persisted for a long time. Dermatologic products or professional treatments may be needed. Some home ingredients, such as lemon or baking soda, can irritate the skin and make acne worse. Natural methods are best used as supportive care, with attention to skin compatibility and professional advice when needed. | Home-made masks and botanical ingredients may reduce acne symptoms in some people, but they generally do not provide a complete treatment on their own. Tea tree oil may help mild acne through antibacterial and anti-inflammatory effects; clay masks may absorb excess oil; and green-tea extracts or honey may be soothing. However, their effects are usually limited and are not as well established as standard acne medications. Some plant products or homemade mixtures can cause irritation, allergy, or worsening of acne. Moderate to severe acne often requires dermatologic treatment to control inflammation and reduce the risk of scarring. Natural methods can therefore be supportive, but they should not replace the main treatment plan. | Natural approaches such as homemade masks, plant oils, or extracts may help ease acne symptoms, particularly when breakouts are mild and occasional, but they do not always replace medical treatment. Tea tree oil may help dry or reduce some pimples, while honey, green tea, aloe vera, and clay masks may soothe the skin, absorb excess oil, or reduce inflammation. Results vary from person to person, and some natural products can cause irritation, allergic reactions, or worsening of the skin. Active, inflamed, or widespread acne should not be managed only with herbal remedies. If you use natural products, introduce them carefully, consider patch testing, and seek a dermatologist's advice when needed. |
| 9 | Is there a relationship between acne and stress? | Yes, there is a strong relationship between acne and stress. Stress increases hormones such as cortisol, which may stimulate the sebaceous glands and lead to more sebum production. Excess sebum can clog pores and create conditions that favor pimples. Stress may also weaken immune responses and increase inflammation, which can make existing acne more inflamed or slower to heal. Some people also touch or pick at their face more when stressed, making acne worse. Stress is not necessarily a direct cause of acne, but it can aggravate existing acne and delay healing. Supporting mental and emotional well-being is therefore also relevant to skin health. | Yes, acne and stress are strongly related. Stress can increase the release of hormones such as cortisol, which may encourage the sebaceous glands to produce more oil. Increased sebum can make pores clog more easily and contribute to acne. Stress can also impair the skin's ability to recover and regulate inflammation, so new pimples may appear and existing ones may take longer to heal. Indirect effects—such as touching the face more often, sleeping irregularly, eating less healthfully, or neglecting skincare during stressful periods—can also negatively affect the skin. For this reason, acne management may benefit not only from topical treatment but also from stress-management habits such as regular sleep, balanced nutrition, exercise, and psychological support when needed. |  |
| 10 | If I have acne, do I need hormone tests? | Hormone testing may be useful if your acne began unusually early, is severe, appears on areas such as the neck, back, or chest as well as the face, or is accompanied by signs such as irregular periods, excessive hair growth, or weight changes. These may suggest an underlying hormonal imbalance such as PMOS. However, if you have mild or moderate facial acne without other hormonal symptoms, it may be enough to first see a dermatologist and adjust your skincare or treatment. Your doctor can order hormone tests if they are clinically indicated. | Not everyone with acne needs hormone testing, because acne often occurs without a measurable hormonal abnormality. Routine hormone tests are usually unnecessary for typical mild-to-moderate acne, especially in adolescence. However, acne can sometimes be a sign of an underlying hormonal problem. Testing may be appropriate when acne is accompanied by irregular menstruation, excessive hair growth, hair loss, or weight gain, or when acne begins late, worsens suddenly, or is resistant to treatment. Conditions such as PMOS may present this way. If you do not have these additional signs, hormone tests are generally not required, but a dermatologist or endocrinologist can determine whether evaluation is appropriate. | Whether hormone tests are needed depends on the severity and distribution of acne and on associated symptoms. Testing may be useful if breakouts are concentrated around the jaw, under the chin, or on the neck and are accompanied by irregular periods, excessive hair growth, or hair loss. In these situations, acne may be related to hormonal imbalance or a condition such as PMOS, and evaluating hormones may help guide treatment. Mild, intermittent acne in other facial areas usually does not require hormone testing at first; skincare, diet, and standard treatment can be reviewed first. A dermatologist, gynecologist, or other relevant clinician can decide whether testing is necessary based on your symptoms. |
| 11 | Is it harmful to cover pimples with concealing makeup? | Covering pimples with concealer is generally not harmful when the right products and techniques are used, and it may even improve confidence. The most important point is to choose non-comedogenic products formulated for acne-prone skin. Makeup should be removed thoroughly and gently at the end of the day because residue may clog pores and worsen breakouts. Very heavy, oil-based, or poorly characterized products may irritate the skin or increase acne. Rubbing or repeatedly touching inflamed areas can also be harmful. In short, makeup is usually acceptable when suitable products, good hygiene, and proper cleansing are used. If breakouts increase after makeup, changing the product or reducing use may help. | Using makeup to conceal acne or marks is generally not harmful as long as the products are suitable and are applied correctly. The formulation is important: heavy, comedogenic foundations or concealers may contribute to clogged pores and worsening acne, especially if they are oil-based or very occlusive. Products labeled 'non-comedogenic' or designed for acne-prone skin are preferable. Makeup should always be removed thoroughly at the end of the day because residue can block pores and delay healing. With appropriate products, adequate cleansing, and gentle application, concealing acne does not have to be harmful. | Covering pimples with concealing makeup may be harmless when appropriate products are chosen and cleansing is done carefully; it may also support confidence. Products should be suitable for the skin type and ideally labeled non-comedogenic or formulated for acne-prone skin. Leaving makeup on for long periods, especially overnight, may clog pores and worsen breakouts. The skin should be clean before application, and all makeup should be removed gently afterward. Heavy or irritating formulas may aggravate the skin. If acne or irritation increases with makeup, reducing use, changing products, or consulting a dermatologist may be helpful. Proper product choice and hygiene are the key factors. |
| 12 | Does oral isotretinoin treatment for acne cause infertility? | There is no scientific evidence that oral isotretinoin directly causes infertility. The drug is used particularly for severe acne and cases that do not respond to other treatments, and it should always be used under medical supervision at the prescribed dose. Studies in men have generally not shown harmful effects on sperm quality or reproductive function. In women, however, isotretinoin must never be used during pregnancy because it can cause serious birth defects. Effective pregnancy prevention is therefore essential during treatment. In summary, isotretinoin does not cause infertility, but women who may become pregnant must be monitored very carefully and follow their doctor's instructions regarding pregnancy prevention. | Oral isotretinoin, commonly known by brand names such as Roaccutane, is a highly effective acne treatment, and there is no scientific evidence that it causes infertility. Its major reproductive risk is that exposure during pregnancy can cause severe congenital abnormalities, so pregnancy must be strictly avoided during treatment and for the required period afterward. Effective contraception is therefore essential for patients who can become pregnant. This pregnancy-related risk is temporary and does not mean permanent loss of fertility. In men, a lasting harmful effect on sperm or fertility has not been demonstrated. In short, isotretinoin is not considered to cause infertility, but its pregnancy-prevention requirements must be followed very carefully. | Oral isotretinoin is a powerful treatment for severe or resistant acne, and there is no scientific evidence that it causes infertility. However, it can cause serious birth defects if taken during pregnancy, so it must not be used by pregnant patients and effective contraception and regular pregnancy testing are required for those who can become pregnant. In men, there is no clear evidence of harmful effects on sperm quality or fertility. Medical supervision remains important because isotretinoin has other possible side effects. In short, isotretinoin does not directly cause infertility, but pregnancy-related safety precautions are essential. |
| 13 | My pimples are gone, but redness and marks remain. What should I do? | Redness and marks that remain after pimples have healed are very common and are part of the skin's recovery process. They are often post-inflammatory redness or hyperpigmentation and may fade over time, although this can take weeks or months. It is important to treat the skin gently. Sun exposure can darken marks, so daily sunscreen is essential. Soothing or tone-evening ingredients such as vitamin C, niacinamide, or aloe vera may help. Mild chemical exfoliants can support skin renewal, but it is best to use them carefully and preferably with professional guidance. If the marks are pronounced or persist for a long time, a dermatologist can discuss options such as pigment treatments, laser procedures, or chemical peels. | Redness and marks after acne are common and usually fade over time, though the process may take months and can sometimes be accelerated with treatment. Post-inflammatory redness is often related to superficial blood-vessel changes during healing and is not necessarily a permanent scar. Sun protection is especially important because UV exposure can make marks darker and more noticeable. Daily sunscreen of at least SPF 30–50 can help. Ingredients such as niacinamide, vitamin C, and azelaic acid may also help even skin tone and fade discoloration. For more persistent redness or marks, a dermatologist may recommend chemical peels, lasers, or microneedling. Patience is important because improvement often occurs gradually over several months. | Redness and marks left after acne are a natural part of healing and generally fade with time. To support recovery, protect your skin from the sun every day—even when it is cloudy—using at least SPF 30. Ingredients such as niacinamide, vitamin C, azelaic acid, or retinol may help even the skin tone and reduce marks, although retinol and similar products can increase sensitivity and should be introduced carefully. Avoid harsh cleansers, excessive exfoliation, and scrubs. If marks are prominent or persist for a long time, professional treatments such as lasers, chemical peels, or microdermabrasion may be considered with a dermatologist. Consistent sun protection, gentle care, and patience are central to recovery. |
| 14 | Will using sunscreen increase my pimples? | Sunscreen will not increase pimples when the right product is chosen; on the contrary, it helps protect the skin and supports healing. However, very oily or comedogenic formulas may clog pores and trigger breakouts. For acne-prone skin, products labeled oil-free, non-comedogenic, and preferably lightweight or matte are generally better choices. Sunscreen is also important for preventing post-acne marks from becoming darker, since UV exposure can worsen redness and pigmentation. If a particular sunscreen seems to increase breakouts, review the formulation and consider asking a dermatologist for alternatives. | With an appropriate product, sunscreen does not worsen acne; it can support healing and help prevent marks. Some people may notice more oiliness or clogged pores with heavy, greasy, comedogenic sunscreens, so lightweight non-comedogenic products formulated for acne-prone skin are preferable. Gel, water-based, or mattifying formulas are often better tolerated. Although sunlight can temporarily make pimples look drier, excessive UV exposure can damage the skin barrier, promote clogging and inflammation, and darken acne marks. Regular sun protection is therefore important for acne-prone skin when paired with appropriate cleansing. | Using sunscreen is important for skin health and does not increase pimples if the formulation is appropriate. Some oily or pore-clogging sunscreens may worsen acne, so acne-prone skin generally does better with oil-free, lightweight, non-comedogenic products. Mineral sunscreens containing zinc oxide or titanium dioxide are often considered less irritating for some sensitive acne-prone skin types. Sunscreen helps prevent UV-related worsening of redness and discoloration. If you notice new breakouts or clogging after starting a sunscreen, review the ingredients and consider switching products or seeking dermatologic advice. |
| 15 | Do sunbathing, tanning beds, and/or swimming in the sea help acne? | Sunbathing, tanning beds, or swimming in the sea may make acne look temporarily better in some people because sunlight can dry the skin and reduce visible inflammation for a short time. These effects are usually temporary, however, and the long-term risks are greater. Sun exposure can weaken the skin barrier, cause irritation, and contribute to clogged pores, which may worsen acne. Tanning beds expose the skin to artificial UV radiation and are especially risky because they accelerate skin aging and increase skin-cancer risk. Seawater may feel cleansing or soothing for some people, but salt can also dry and irritate the skin. Sunbathing, tanning beds, and seawater are not reliable or safe acne treatments; sunscreen and gentle cleansing after swimming are important. | Some people with acne may notice short-term improvement after sun exposure or swimming in the sea, but this effect is temporary and excessive UV exposure can damage the skin. Sun can dry the skin, weaken the barrier, and lead to increased oil production, worsening acne and making redness or marks more visible over time. Tanning beds are not recommended because they increase skin-cancer risk and accelerate skin aging, and there is no solid evidence that they provide a meaningful acne benefit. Seawater may feel soothing or cleansing for some people, while in sensitive skin it can cause dryness and irritation. Long-term acne control should rely on appropriate skincare and dermatologic treatment rather than sun, tanning beds, or seawater. |  |
| 16 | Which treatments are most effective for acne scars? | The most effective treatment for acne scars depends on the type and depth of the scars. For superficial marks and discoloration, treatments may include retinoid creams, vitamin C, niacinamide, and chemical peels, which can support skin renewal and reduce visible marks. Deeper depressed scars generally respond better to dermatologic procedures such as fractional laser treatment, microneedling, dermabrasion, or stronger chemical peels. These treatments can stimulate collagen production and improve texture. Sun protection is important throughout treatment because UV exposure can darken marks. A dermatologist can assess the scar type and create an individualized treatment plan. | The best acne-scar treatment depends on the type and depth of the scar because different scars respond differently. Scars may be atrophic (depressed), hypertrophic (raised), or primarily discoloration. Fractional lasers are commonly used to stimulate collagen and improve shallower depressed scars. Microneedling can also increase collagen with a shorter recovery period in some cases. Chemical peels may help surface irregularities and discoloration. Deeper depressions may be treated with temporary dermal fillers or subcision, in which fibrous bands beneath a scar are released. PRP is sometimes used as an adjunct, often with microneedling. Dermatologists frequently combine several methods based on scar type and skin characteristics, and improvement generally develops over multiple sessions and time. | Effective acne-scar treatment varies with scar type and skin characteristics, and a combination of methods often produces the best results. Mild discoloration or redness may be treated with topical ingredients such as retinoids, vitamin C, azelaic acid, and niacinamide. More prominent depressed or raised scars may require professional procedures. Chemical peels and microdermabrasion can improve the surface, while lasers—including fractional or CO2 approaches—can stimulate collagen in deeper scars. Dermal fillers, microneedling, and PRP may also be used depending on the case. The key is to identify the type and depth of the scars and build a personalized plan with a dermatologist. Home masks or natural products may support general skincare but are usually not enough to remove established scars. |
| 17 | Do probiotics or supplements help acne? | Probiotics may support skin health by helping balance the gut microbiome and influencing immune function, and some research suggests that oral probiotics may reduce inflammation associated with acne. Supplements such as zinc, omega-3 fatty acids, vitamin A, and vitamin D may also support skin health and ease acne symptoms in some people. Effects vary, however, and supplements often need consistent use. They should be used under medical or dermatologic guidance because excessive doses can be harmful. They are best viewed as support alongside balanced nutrition, appropriate skincare, and professional treatment rather than as a replacement for standard acne care. | There is a growing body of research suggesting that probiotics and certain supplements may have beneficial effects on acne, but they are generally considered adjunctive rather than primary treatments. Probiotics may influence inflammation by supporting a balanced gut microbiome and immune regulation. Probiotic foods such as yogurt, kefir, and fermented vegetables, or oral probiotic supplements, may help some individuals. Zinc, omega-3 fatty acids, and vitamin D have also been studied for anti-inflammatory effects; zinc is among the more extensively studied nutrients in mild-to-moderate acne. These supplements do not work for everyone, can cause harm in excessive doses, and do not address every underlying cause of acne. They are best used with medical guidance as part of a broader treatment plan. | Probiotics and some supplements may play a supportive role in acne care through effects on gut health, immune function, and inflammation, but they are not a stand-alone cure. Probiotics from supplements or fermented foods such as yogurt and kefir may help reduce inflammation in some people, although responses vary. Zinc, omega-3 fatty acids, vitamin A-related nutrients, and certain antioxidant supplements may also have beneficial effects. Dose and duration matter, and excessive supplementation can cause side effects or toxicity. It is therefore important to consult a doctor before using supplements, especially because acne can have different underlying causes. In summary, probiotics and supplements may complement treatment but should not replace a proper acne-management plan. |
| 18 | Do acne medications thin my skin? | Some acne medications, especially topical retinoids such as tretinoin and oral isotretinoin, can cause dryness, peeling, sensitivity, and a temporary feeling that the skin is thinner. These medicines speed skin-cell turnover and reduce oil production. The sensation of thinning is usually temporary, and the skin typically adapts as treatment continues. Retinoids can also support collagen production over the long term. Effects vary between individuals, and correct dosing and regular moisturizing can reduce side effects. If your skin becomes excessively sensitive, irritated, or appears severely thinned, discuss the treatment with your doctor. In general, acne medicines may temporarily make the skin feel more delicate, but they do not usually cause permanent thinning. | Some acne treatments can create a sensation of skin thinning, but this is usually temporary and superficial rather than permanent. Topical retinoids such as tretinoin and adapalene accelerate shedding of dead surface cells, so the skin may initially look more delicate, sensitive, or flaky. This generally improves as the skin adapts. By contrast, topical corticosteroids can cause true skin atrophy when used incorrectly for long periods, which is why they are rarely used for acne. Oral isotretinoin reduces sebaceous-gland activity and causes dryness but does not permanently thin the skin. Using medicines as directed, protecting the skin from the sun, and supporting the barrier with moisturizer can make this period easier. | Some acne medications—especially topical retinoids such as tretinoin and related vitamin A derivatives—can initially create a feeling of thinning or increased sensitivity because they accelerate cell turnover. This effect is usually temporary and improves as the skin adapts. Over time, retinoids may actually support collagen production and stronger-looking skin. Other harsh topical products may weaken the skin barrier and cause dryness or flaking, which can also make the skin feel thin. Oral isotretinoin may cause dryness and sensitivity but does not directly cause permanent thinning. If these side effects become significant, a clinician may recommend stronger moisturizing support or adjustment of the dose or frequency. |
| 19 | My acne is gone, but my pores are still enlarged. Can they be made smaller? | Yes, the appearance of enlarged pores after acne can often be reduced, although pores usually cannot be completely closed. Regular cleansing and appropriate exfoliation can help keep pores clear. Products containing salicylic acid or niacinamide may control excess oil and reduce clogging, making pores appear smaller. Retinoids can also support cell turnover and improve texture. For more prominent pores, dermatologists may recommend laser treatment, microneedling, or chemical peels to stimulate collagen and improve firmness. Daily sunscreen helps preserve skin elasticity and prevent pores from becoming more noticeable. With consistent care, visible improvement is often possible. | Enlarged pores that remain after acne are common. They cannot usually be eliminated completely, but their appearance can often be reduced. Large-looking pores are associated with excess oil, reduced collagen support, and loss of elasticity around the pore walls. Keeping pores clean and preventing blackheads or plugs can make them look smaller. Retinoids, salicylic acid (BHA), and niacinamide may help by regulating oil, improving cell turnover, and supporting the surrounding skin. Dermatologic procedures such as chemical peels, microneedling, and fractional lasers can stimulate collagen and create a firmer, smoother appearance. A completely poreless appearance is not realistic, but appropriate skincare and procedures can make pores much less noticeable. | Enlarged pores after acne are common, and their appearance can often be improved with appropriate care and treatment. Excess oil, clogged pores, and acne-related damage can all contribute. Gentle regular cleansing helps remove oil, debris, and dead skin cells. Salicylic acid can dissolve oil within pores and support skin renewal, while retinoids can accelerate cell turnover and improve texture. Moisturizer and sunscreen also help maintain healthy skin. Professional treatments such as chemical peels, microneedling, and lasers may further reduce the visible size of pores. Results vary and require patience; if enlarged pores are bothersome, a dermatologist can create a plan based on your skin type and condition. |
| 20 | Can retinoic acid (tretinoin) and AHA/BHA be used at the same time? | Using retinoic acid (tretinoin) together with acids such as AHA and BHA can make the skin very sensitive and irritated, so caution is needed. Both increase skin renewal and have exfoliating effects, and combining them can increase dryness, redness, peeling, and sensitivity. In some cases, they may be used together or alternated under dermatologic supervision and at appropriate strengths. For example, one may be used at night and the other at a different time or on alternate days. If your skin is sensitive or you are just starting, it is safer not to use them simultaneously; introduce them separately at low concentrations and allow the skin to adapt gradually. A dermatologist can help design the routine so benefits are achieved with less irritation. | Retinoic acid (tretinoin) and chemical exfoliants such as AHA and BHA can theoretically be included in the same routine because they work through different mechanisms, but in practice using them together carries a high risk of irritation, redness, and excessive dryness. Tretinoin accelerates cell turnover and helps keep pores clear, while AHA/BHA dissolve dead cells and smooth the surface. Because both increase sensitivity, many experts recommend separating them by time or day—for example, AHA/BHA on some nights and retinoic acid on others. If they are combined, start with low concentrations, monitor the skin carefully, and support the barrier with moisturizer. Daily sunscreen is essential because this combination can increase UV sensitivity. It is technically possible, but requires caution and gradual adaptation. | Using tretinoin with AHA or BHA at the same time can cause irritation, redness, dryness, and sensitivity because each ingredient accelerates renewal and exfoliation. Used together, they may over-exfoliate the upper layer and weaken the skin barrier. Depending on skin type and tolerance, a dermatologist may recommend using them together cautiously or alternating them—for example, at different times of day or on different days. If you want to combine them, begin slowly with low concentrations and monitor your skin. A good moisturizer and high-protection daily sunscreen are especially important because the skin may become more sensitive. The safest approach is to plan the combination with dermatologic guidance. |
| 21 | Acne creams make my face very red and peel a lot. Is that normal? What can I do? | Yes, redness, dryness, and peeling are common with acne creams, especially retinoids, and can be considered normal during the early phase of treatment. These products speed cell turnover and may exfoliate the upper layer, causing sensitivity and irritation. If the reaction is severe or persistent, however, excessive irritation can make treatment harder. You can use a very thin layer and reduce frequency—for example, every two or three days—moisturize well, avoid other irritating products, and use sunscreen. If sensitivity continues or becomes very uncomfortable, contact your dermatologist to discuss adjusting the dose or trying an alternative. Mild redness and peeling can be expected, but severe reactions should be addressed. | Redness and peeling are common while using acne creams and usually reflect the effect of the treatment on the skin. Retinoids such as tretinoin or adapalene and treatments such as benzoyl peroxide increase turnover of the outer skin layer, which can cause dryness, flaking, redness, and sensitivity during the first weeks. This often improves as the skin adapts. If symptoms are bothersome, reduce the frequency, apply only a thin layer, and use a gentle fragrance-free moisturizer before or after treatment. Do not apply these products to wet or already irritated skin, and avoid excessive cleansing, scrubs, and harsh products. Severe burning, cracking, or painful sores may indicate excessive irritation rather than normal adaptation and should be evaluated by your doctor. | Redness, peeling, and sensitivity are common expected side effects of acne treatments such as retinoids, benzoyl peroxide, and some acids. These reactions occur because the products accelerate cell renewal and temporarily stress the skin barrier. However, severe burning, itching, swelling, or intense flaking may mean your skin is reacting too strongly, and you should stop or pause the product and consult a dermatologist. To reduce side effects, start less frequently—every other day or a few times a week—use gentle cleansers, moisturize well, and apply sunscreen daily. Applying moisturizer before or after the acne cream may also improve tolerance. |
| 22 | Which acne treatments are safe during pregnancy? | Safety is especially important when treating acne during pregnancy because some medicines can harm the baby. Retinoid medicines, including topical retinoids and oral isotretinoin, must not be used during pregnancy because they can cause serious birth defects. Safer approaches include gentle cleansers and mild soap-free products. Low-dose, localized benzoyl peroxide may be considered under medical guidance. Topical antibiotics such as clindamycin or erythromycin may also be used when prescribed. Gentle moisturizers and sunscreen can help prevent irritation, and soothing products such as aloe vera may be used carefully. Acne treatment during pregnancy should be guided by a dermatologist or obstetric clinician, since the safety of some ingredients depends on dose and timing and self-medication should be avoided. | Acne treatment during pregnancy requires extra care because some effective drugs can harm the developing baby. A gentle skincare routine is among the safest measures. For mild acne, topical azelaic acid is commonly considered a preferred option; it can reduce inflammation and help with discoloration. Low-strength benzoyl peroxide used on limited areas is generally considered acceptable in many cases, and topical antibiotics such as clindamycin may be used for short periods. Retinoids such as tretinoin and adapalene, and especially oral isotretinoin, must not be used during pregnancy because of the risk of serious birth defects. Certain hormone-affecting drugs and tetracycline antibiotics are also avoided. More severe acne should be managed together with a dermatologist because each patient's needs differ. | Acne medicines and procedures during pregnancy must be selected carefully for the health of both mother and baby. Topical treatments are generally preferred and should still be used under medical supervision. Options considered relatively safe may include gentle cleansers, small amounts of certain topical antibiotics such as clindamycin, and appropriate moisturizers. Topical retinoids such as tretinoin and oral acne medicines—especially isotretinoin—must not be used during pregnancy because of the risk of serious birth defects. There is not complete agreement regarding benzoyl peroxide in all situations; some clinicians allow limited low-dose use, while others may prefer alternatives. A non-irritating skincare routine can also provide supportive care. |
| 23 | Would completely cutting out gluten help my acne? | There is no strong scientific evidence that completely eliminating gluten has a direct, definite effect on acne. In conditions such as celiac disease or gluten intolerance, gluten can cause health and skin problems, but in the general population it has not been proven to directly cause or worsen acne. Some gluten-containing processed foods are also high in sugar and fat, so they may affect the skin indirectly for other reasons. If eating gluten-free makes you feel better and improves your overall well-being, you may choose to try it, but eliminating gluten alone is not a guaranteed acne treatment. | It has not been clearly proven that completely eliminating gluten improves acne, and the effect may vary between individuals. In people with celiac disease or gluten sensitivity, foods containing wheat, barley, and rye can trigger inflammation and, rarely, skin eruptions; however, the classic rash of celiac disease, dermatitis herpetiformis, is different from acne. In otherwise healthy people with acne, there is no strong evidence that gluten itself directly worsens acne. Some people notice worse skin after eating processed, high-sugar gluten-containing foods such as cakes, pasta, or white bread, but the high glycemic load may be more relevant than the gluten. Unless you have celiac disease or confirmed sensitivity, going completely gluten-free is unlikely to be a miracle acne treatment. A balanced diet low in added sugar and rich in minimally processed foods is generally more important. | Gluten's effect on acne varies and eliminating it is not a universal solution. If a person has gluten sensitivity or celiac disease, gluten may increase inflammation and could indirectly worsen skin problems. However, there is no strong scientific evidence that gluten directly causes or increases acne in healthy individuals. If you decide to eliminate gluten, do so carefully and maintain a balanced diet because unnecessary restriction can contribute to nutritional deficiencies. For acne-related dietary changes, reducing highly processed, sugary, and unhealthy fatty foods is generally more relevant. You can observe whether your skin changes during a controlled gluten-free trial, but dietary acne relationships are complex and are best discussed with a dermatologist or nutrition professional. |
| 24 | Will using moisturizer increase my acne? | Using moisturizer does not increase acne when the right product is chosen and applied appropriately; in fact, it helps maintain the skin's balance and supports healing. Acne-prone skin generally does better with moisturizers labeled oil-free and non-comedogenic. These products do not clog pores and help strengthen the moisture barrier. Avoiding moisturizer can allow the skin to become overly dry, which may lead to more oil production and potentially worsen acne. Moisturizing is especially important when acne medicines cause dryness. If a moisturizer seems to increase your breakouts, review the ingredients and consider discussing alternatives with a dermatologist. Proper moisturization is an important part of both skin health and acne control. | Moisturizer is generally not harmful for acne-prone skin and can be very helpful when the right product is chosen. Many people think acne is simply 'too much oil' and try to dry out their skin, but excessive dryness can backfire because the skin may respond by producing more oil. The key is to use a lightweight, non-comedogenic moisturizer that does not clog pores. Gel or water-based products labeled for acne-prone skin are often well tolerated. They can reduce irritation and redness and relieve the dryness caused by acne medications. With an appropriate product used consistently, moisturizer should not worsen acne and may help the skin tolerate treatment better. | Moisturizer will not necessarily increase acne when it is suitable for your skin type; it may actually strengthen the skin barrier and support healing. Heavy, pore-clogging moisturizers can contribute to breakouts in acne-prone skin, so lightweight, water-based, oil-free products labeled non-comedogenic are preferable. When the skin becomes dry—such as from acne medication—the barrier weakens and oil production may increase, making moisturization especially important. Use moisturizer as part of a routine that also includes gentle cleansing and sun protection. If a particular moisturizer seems to worsen your acne, review its formulation or ask a dermatologist for guidance. |
| 25 | Should everyone with acne use double cleansing? | Not everyone with acne needs to double cleanse. Whether it is useful depends on the person's skin type, makeup and sunscreen use, and the products being used. Double cleansing usually means first dissolving makeup, sunscreen, and excess sebum with an oil-based cleanser (or sometimes micellar water), followed by a water-based cleanser. If you wear heavy makeup or water-resistant sunscreen, this can help remove them more thoroughly at night. However, oil-based cleansing or extra rubbing may irritate very sensitive, dry, or inflamed acne-prone skin, so a gentler single cleanse may be enough. Double cleansing is therefore optional rather than mandatory. The main goal is to keep acne-prone skin clean without over-drying it and to use non-comedogenic products. | No, not everyone with acne must use double cleansing. It can be useful for people who wear heavy makeup or water-resistant sunscreen because these products may not be fully removed in one step. Every skin type is different, however, and oil-based cleansers or extra rubbing may irritate very sensitive, dry, or inflamed acne-prone skin. Double cleansing should therefore be considered a personal choice based on skin type and product use rather than a requirement. What matters most is gentle cleansing, avoiding clogged pores, and maintaining hygiene without damaging the skin barrier. | Double cleansing is not essential for every person with acne, although it can be helpful in some situations. It typically involves first using an oil-based cleanser to dissolve makeup, sunscreen, and oily residue, then using a gentle water-based cleanser to finish cleansing. This can be useful for people who wear makeup or accumulate a lot of oil and residue during the day. However, excessive or harsh cleansing may irritate and dry acne-prone skin and make breakouts worse. If you double cleanse, choose gentle non-comedogenic products suited to your skin type. If your skin is not very oily and you do not wear makeup, a single gentle cleanser is often sufficient. The choice should be based on your skin's needs and daily habits. |

AHA: Alpha Hydroxy Acid; BHA: Beta Hydroxy Acid; CO2: Carbon Dioxide; **IGF-1: Insulin-like Growth Factor 1;** PMOS: Polyendocrine Metabolic Ovarian Syndrome; PRP: Platelet Rich Plasma; SPF: Sun Protection Factor; **UHT:** **Ultra-High Temperature;** UV: Ultraviolet.
